# Supplementary material for: Does the Locally-Adaptive Model of Archaeological Potential (LAMAP) work for hunter-gatherer sites? A test using data from the Tanana Valley, Alaska
Source: PLoS One. 2022 Mar 17;17(3):e0265597. doi: 10.1371/journal.pone.0265597 (PMC8929620; doi:10.1371/journal.pone.0265597)
Supplement: S1 Table — Data from the Alaska Heritage Resources Survey, the State of Alaska’s Office of History and Archaeology. These sites were used to build and test LAMAP models in the Tanana valley. Locational information for each site was made available for the purposes of research, but locational data are redacted here in order to protect heritage resources. (DOCX) [file pone.0265597.s001.docx]

**S1 Table. AHRS sites database**

Data from the Alaska Heritage Resources Survey, the State of Alaska’s Office of History and Archaeology. These sites were used to build and test LAMAP models in the Tanana valley. Locational information for each site was made available for the purposes of research, but locational data are redacted here in order to protect heritage resources.

| **Site** | **Components** | **Stratified** | **Pre-10K BP** | **10–5K BP** | **5K BP - Present** | **Date Unknown** |
| --- | --- | --- | --- | --- | --- | --- |
| **HEA_00102** | Single | No |  |  |  | X |
| **HEA_00103** | Single | No |  |  |  | X |
| **HEA_00104** | Single | No |  |  |  | X |
| **HEA_00685** | Single | No |  |  |  | X |
| **XBD_00010** | Single | No |  |  |  | X |
| **XBD_00011** | Single | No |  |  |  | X |
| **XBD_00012** | Single | No |  |  |  | X |
| **XBD_00013** | Single | No |  |  |  | X |
| **XBD_00014** | Single | No |  |  |  | X |
| **XBD_00015** | Single | No |  |  |  | X |
| **XBD_00017** | Single | No |  |  |  | X |
| **XBD_00018** | Single | No |  |  |  | X |
| **XBD_00019** | Single | No |  |  |  | X |
| **XBD_00028** | Multi | Yes | X |  |  |  |
| **XBD_00031** | Single | No |  |  |  | X |
| **XBD_00042** | Single | No |  |  |  | X |
| **XBD_00071** | Multi | No |  |  |  | X |
| **XBD_00072** | Single | No |  |  |  | X |
| **XBD_00073** | Single | No |  |  |  | X |
| **XBD_00089** | Single | No |  |  |  | X |
| **XBD_00106** | Multi | No |  |  | X |  |
| **XBD_00107** | Single | No |  |  |  | X |
| **XBD_00108** | Single | No |  |  |  | X |
| **XBD_00109** | Single | No |  |  |  | X |
| **XBD_00110** | Single | No |  |  | X |  |
| **XBD_00131** | Multi | Yes | X | X | X |  |
| **XBD_00155** | Multi | Yes | X | X |  |  |
| **XBD_00156** | Multi | Yes | X | X | X |  |
| **XBD_00157** | Single | No |  |  |  | X |
| **XBD_00158** | Single | No |  |  |  | X |
| **XBD_00159** | Single | Yes |  |  | X |  |
| **XBD_00160** | Single | No |  |  |  | X |
| **XBD_00161** | Single | No |  |  |  | X |
| **XBD_00165** | Single | No |  |  |  | X |
| **XBD_00166** | Single | No |  |  |  | X |
| **XBD_00167** | Single | No |  |  |  | X |
| **XBD_00171** | Multi | Yes |  |  |  | X |
| **XBD_00183** | Multi | Yes |  |  | X |  |
| **XBD_00235** | Single | No |  |  | X |  |
| **XBD_00247** | Single | No |  |  |  | X |
| **XBD_00265** | Single | No |  |  |  | X |
| **XBD_00283** | Single | No |  |  | X |  |
| **XBD_00286** | Single | No |  |  | X |  |
| **XBD_00287** | Single | No |  |  | X |  |
| **XBD_00288** | Single | No |  | X |  |  |
| **XBD_00289** | Single | No | X |  |  |  |
| **XBD_00290** | Single | No | X |  |  |  |
| **XBD_00291** | Multi | No | X |  |  |  |
| **XBD_00297** | Single | No | X |  |  |  |
| **XBD_00298** | Multi | Yes | X |  |  |  |
| **XBD_00299** | Single | No |  |  |  | X |
| **XBD_00300** | Single | No |  |  |  | X |
| **XBD_00301** | Single | No |  |  | X |  |
| **XBD_00302** | Single | No |  |  |  | X |
| **XBD_00303** | Single | No |  | X |  |  |
| **XBD_00304** | Single | No |  |  |  | X |
| **XBD_00305** | Single | No |  |  |  | X |
| **XBD_00306** | Single | No |  |  |  | X |
| **XBD_00307** | Single | No |  | X |  |  |
| **XBD_00308** | Single | No | X |  |  |  |
| **XBD_309** | Single | No |  |  |  | X |
| **XBD_00311** | Single | No |  | X |  |  |
| **XBD_00312** | Single | No |  | X |  |  |
| **XBD_00313** | Single | No |  | X |  |  |
| **XBD_00314** | Single | No |  |  |  | X |
| **XBD_00315** | Single | No |  |  |  | X |
| **XBD_00316** | Single | No |  |  | X |  |
| **XBD_00317** | Single | No |  | X |  |  |
| **XBD_00318** | Single | No |  |  |  | X |
| **XBD_00319** | Single | No |  |  |  | X |
| **XBD_00320** | Single | No |  |  |  | X |
| **XBD_00321** | Single | No |  |  |  | X |
| **XBD_00322** | Single | Yes |  |  |  | X |
| **XBD_00323** | Single | Yes |  |  |  | X |
| **XBD_00324** | Single | No |  |  | X |  |
| **XBD_00325** | Single | No |  | X |  |  |
| **XBD_00326** | Single | No |  | X |  |  |
| **XBD_00327** | Single | No |  |  |  | X |
| **XBD_00328** | Single | No |  |  | X |  |
| **XBD_00335** | Multi | Yes |  |  | X |  |
| **XBD_00338** | Multi | Yes | X |  |  |  |
| **XBD_00339** | Multi | Yes |  | X | X |  |
| **XBD_00340** | Single | No |  | X |  |  |
| **XBD_00341** | Single | No |  | X |  |  |
| **XBD_00342** | Single | No |  |  | X |  |
| **XBD_00343** | Single | No |  |  |  | X |
| **XBD_00344** | Single | Yes |  |  | X |  |
| **XBD_00345** | Single | No |  |  |  | X |
| **XBD_00361** | Single | No |  |  | X |  |
| **XBD_00362** | Single | No |  |  | X |  |
| **XBD_00363** | Single | No | X |  |  |  |
| **XBD_00371** | Single | No |  |  |  | X |
| **XBD_00374** | Single | No |  |  |  | X |
| **XBD_00377** | Single | No |  |  |  | X |
| **XBD_00378** | Single | No |  |  |  | X |
| **XBD_00383** | Single | No |  |  |  | X |
| **XBD_00389** | Single | No |  |  |  | X |
| **XBD_00390** | Single | No |  |  |  | X |
| **XBD_00391** | Single | No |  |  |  | X |
| **XBD_00392** | Single | No |  |  |  | X |
| **XBD_00393** | Single | No |  |  |  | X |
| **XBD_00407** | Single | No |  |  |  | X |
| **XBD_00410** | Single | No |  |  |  | X |
| **XBD_00411** | Single | No |  | X |  |  |
| **XBD_00412** | Single | No |  |  | X |  |
| **XBD_00413** | Single | No |  |  |  | X |
| **XBD_00415** | Single | No |  |  |  | X |
| **XBD_00416** | Single | No |  |  |  | X |
| **XBD_00417** | Single | No |  |  |  | X |
| **XBD_00418** | Single | No |  |  |  | X |
| **XBD_00419** | Single | No |  |  |  | X |
| **XBD_00421** | Single | No |  |  |  | X |
| **XBD_00422** | Multi | Yes |  |  |  | X |
| **XBD_00425** | Single | No |  |  |  | X |
| **XBD_00426** | Single | No |  |  |  | X |
| **XBD_00427** | Single | No |  |  |  | X |
| **XBD_00428** | Single | No |  |  |  | X |
| **XBD_00429** | Single | No |  |  |  | X |
| **XBD_00430** | Single | No |  |  |  | X |
| **XBD_00431** | Single | No |  |  |  | X |
| **XBD_00444** | Multi | Yes |  | X | X |  |
| **XBD_00445** | Single | No |  |  |  | X |
| **XBD_00448** | Single | No |  |  | X |  |
| **XMH_00232** | Single | No |  |  |  | X |
| **XMH_00233** | Single | No |  |  |  | X |
| **XMH_00234** | Single | No |  |  |  | X |
| **XMH_00235** | Single | No |  |  |  | X |
| **XMH_00236** | Single | No |  |  |  | X |
| **XMH_00237** | Single | No |  |  |  | X |
| **XMH_00299** | Single | No |  |  |  | X |
| **XMH_00300** | Single | No |  |  |  | X |
| **XMH_00301** | Single | No |  |  |  | X |
| **XMH_00302** | Single | No |  |  |  | X |
| **XMH_00303** | Single | No |  |  |  | X |
| **XMH_00304** | Single | No |  |  |  | X |
| **XMH_00305** | Single | No |  |  |  | X |
| **XMH_00306** | Single | No |  |  |  | X |
| **XMH_00307** | Single | No |  |  |  | X |
| **XMH_00310** | Single | No |  |  |  | X |
| **XMH_00313** | Single | No |  |  |  | X |
| **XMH_00829** | Single | No |  |  |  | X |
| **XMH_00830** | Single | No |  |  |  | X |
| **XMH_00831** | Single | No |  |  |  | X |
| **XMH_00832** | Single | No |  |  |  | X |
| **XMH_00833** | Single | No |  |  |  | X |
| **XMH_00834** | Single | No |  |  |  | X |
| **XMH_00835** | Single | No |  |  |  | X |
| **XMH_00836** | Single | No |  |  |  | X |
| **XMH_00837** | Single | No |  |  |  | X |
| **XMH_00839** | Single | No |  |  |  | X |
| **XMH_00840** | Single | No |  |  |  | X |
| **XMH_00841** | Single | No |  |  |  | X |
| **XHM_01414** | Single | No |  |  |  | X |
| **XHM_01415** | Single | No |  |  |  | X |
| **XMH_01434** | Single | No |  |  |  | X |
| **XHM_01435** | Single | No |  |  |  | X |
| **XMH_01436** | Single | No |  |  |  | X |
| **XMH_01437** | Single | No |  |  |  | X |
| **XMH_01438** | Single | No |  |  |  | X |
| **XMH_01439** | Single | No |  |  |  | X |
| **XMH_01440** | Single | No |  |  |  | X |
| **XMH_01441** | Single | No |  |  |  | X |
| **XMH_01442** | Single | No |  |  |  | X |
| **XMH_01443** | Single | No |  |  |  | X |
| **XMH_01444** | Single | No |  |  |  | X |
| **XMH_01445** | Single | No |  |  |  | X |
| **XMH_01446** | Single | No |  |  |  | X |
| **XMH_01447** | Single | No |  |  |  | X |
| **XMH_01448** | Single | No |  |  |  | X |
| **XMH_01449** | Single | No |  |  |  | X |
| **XMH_01450** | Single | No |  |  |  | X |
| **XMH_01451** | Single | No |  |  |  | X |
| **XMH_01452** | Single | No |  |  |  | X |
| **XMH_01453** | Single | No |  |  |  | X |
| **XMH_01454** | Single | No |  |  |  | X |
| **XMH_01491** | Single | No |  |  |  | X |
| **XMH_01492** | Single | No |  |  |  | X |
| **XHM_01544** | Single | No |  |  |  | X |
| **XHM_01545** | Single | No |  |  |  | X |
| **XHM_01549** | Single | No |  |  |  | X |
| **XHM_01550** | Single | No |  |  |  | X |
| **XHM_01551** | Single | No |  |  |  | X |
