# Supplementary material for: Does the Locally-Adaptive Model of Archaeological Potential (LAMAP) work for hunter-gatherer sites? A test using data from the Tanana Valley, Alaska
Source: PLoS One. 2022 Mar 17;17(3):e0265597. doi: 10.1371/journal.pone.0265597 (PMC8929620; doi:10.1371/journal.pone.0265597)
Supplement: S2 Table — These links provide access to scripts used in the LAMAP analysis. (DOCX) [file pone.0265597.s002.docx]

**S2 Table**. **Links to scripts.** These links provide access to scripts used in the LAMAP analysis

| Description | URL |
| --- | --- |
| Direct link to scripts | <https://github.com/wccarleton/lamap_tanana> |
| Repository | https://www.zenodo.org/ |
